# Proteoform Identification Using Multiplexed Top‐Down Mass Spectra

**DOI:** 10.1002/pmic.70020

**Published:** 2025-07-30

**Authors:** Zhige Wang, Xingzhao Xiong, Xiaowen Liu

**Affiliations:** ^1^ Department of Computer Science Tulane University New Orleans Louisiana USA; ^2^ Deming Department of Medicine Tulane University New Orleans Louisiana USA

**Keywords:** database searching, multiplexing, proteoform identification, top‐down proteomics

## Abstract

Top‐down mass spectrometry (TDMS) is the method of choice for analyzing intact proteoforms, as well as their posttranslational modifications and sequence variations. In top‐down tandem mass spectrometry (TD‐MS/MS) experiments, multiple proteoforms are often co‐fragmented, resulting in multiplexed TD‐MS/MS spectra. Due to their increased complexity, compared to spectra from single proteoforms, multiplexed TD‐MS/MS spectra present significant challenges for proteoform identification and quantification. Here we present TopMPI, a new computational tool specifically designed for the identification of multiplexed TD‐MS/MS spectra. Experimental results demonstrate that TopMPI substantially increases the sensitivity and accuracy of proteoform identification in multiplexed TD‐MS/MS spectral analysis compared to existing tools.

**Summary:**

- Top‐down mass spectrometry (TDMS) is a powerful technique for analyzing intact proteoforms; however, identifying multiple co‐fragmented proteoforms from multiplexed tandem mass spectrometry (MS/MS) spectra remains a significant challenge.
- In this paper, we introduce TopMPI, a new computational tool specifically designed to identify multiplexed TD‐MS/MS spectra using a two‐round database search strategy.
- Compared to existing tools, TopMPI significantly improves the sensitivity and accuracy of proteoform identification from multiplexed MS/MS spectra. The development of TopMPI enhances the identification of low abundance proteoforms in complex biological samples and increases the potential of TDMS for discovering proteoform biomarkers in disease studies.

**Abbreviations**
A‐B ratio: additive‐base ratio
ABC: ammonium bicarbonate
ACN: acetonitrile
AGC: automatic gain control
CZE: capillary zone electrophoresis
Da: Dalton
DDA: data‐dependent acquisition
DIA: data‐independent acquisition
DTT: dithiothreitol
EDTA: ethylenediaminetetraacetic acid
FA: formic acid
FDR: false discovery rate
HCD: higher‐energy collisional dissociation
HetM: heterogeneous multiplexed
HomM: homogeneous multiplexed
HPLC: high‐performance liquid chromatography
IPA: isopropanol
LPA: linear polyacrylamide
*m*/*z*
: mass‐to‐charge ratio
MS: mass spectrometry
MS/MS: tandem mass spectrometry
NCE: normalized collision energy
NNMFM: number of normalized matched fragment masses
PBS: phosphate buffered saline
PFPS: precursor‐fragment paired spectrum
PrSM: proteoform‐spectrum match
PSE: precursor selection error
PTM: posttranslational modification
RME: random matching error
RPLC: reverse‐phase liquid chromatography
RPMS: ratio paired multiplexed spectra
SPMS: simple paired multiplexed spectra
TD: top‐down
TDMS: top‐down mass spectrometry

## 1 Introduction

Top‐down mass spectrometry (TDMS) has gained increasing attention in proteomics due to its ability to directly examine and characterize intact proteoforms with various alterations arising from gene mutations, alternative splicing, and posttranslational modifications (PTMs) [1, 2]. Unlike bottom‐up MS, in which combinatorial PTM patterns on proteoforms are lost during enzymatic digestion prior to MS, TDMS enables the analysis of these patterns along with other sequence variations, facilitating the study of their biological functions [3].

In a typical TDMS experiment, MS1 spectra are generated to profile proteoforms in the sample, while tandem mass spectrometry (MS/MS) spectra are acquired to measure fragment ions of proteoforms. Each mass spectrum is represented by a series of peaks, where each peak corresponds to the mass‐to‐charge ratio (*m*/*z*) and intensity of a proteoform ion in an MS1 spectrum or a fragment ion in an MS/MS spectrum. To generate an MS/MS spectrum, an isolation window with a specified *m*/*z* range is applied to isolate and fragment proteoform ions, and the resulting fragment ions are then measured. Ideally, most of the proteoform ions in the isolation window originate from a single proteoform and the resulting MS/MS spectrum is dominated by its fragment ions. However, it is also possible for two or more proteoforms with similar *m*/*z* values to be co‐isolated and co‐fragmented, resulting in a *multiplexed* MS/MS spectrum [3, 4].

In TDMS, multiplexed MS/MS spectra are frequently observed in both data independent acquisition (DIA)‐MS [2] and data dependent acquisition (DDA)‐MS [4], two main MS acquisition strategies. Because DIA‐MS typically uses wider isolation windows than DDA‐MS, multiplexed MS/MS spectra are more frequently encountered in DIA‐MS. Several top‐down DIA‐MS studies were reported recently [4, 5] and demultiplexing methods were employed to analyze top‐down multiplexed DIA‐MS data [4]. However, due to the high complexity of top‐down DIA‐MS data, DDA‐MS remains the predominant method in top‐down proteomics [2]. In this paper, we focus on analyzing multiplexed spectra generated by top‐down DDA‐MS.

Multiplexed top‐down MS/MS spectra can be categorized into two types: homogeneous multiplexed (HomM) MS/MS spectra and heterogeneous multiplexed (HetM) MS/MS spectra. In a HomM‐MS/MS spectrum involving two proteoforms, the two precursor ions typically have the same charge state and differ by only a PTM. Consequently, their molecular mass difference is small (e.g., 15.99 Da due to oxidation), resulting in similar *m*/*z* values and co‐fragmentation. The high similarity between the co‐fragmented proteoforms in a HomM‐MS/MS spectrum leads to many shared fragment ions, making it challenging to accurately distinguish and identify the individual proteoforms [6, 7]. For a HetM‐MS/MS spectrum containing fragment ions from two proteoforms, the fragment ions are typically not shared between the proteoforms. However, in proteoform identification, the fragment ions originating from the second proteoform act as noise when we identify the first proteoform. The presence of these noise ions increases the likelihood of missed or incorrect identifications. In this paper, we focus on the analysis of top‐down HetM‐MS/MS spectra.

There remains a lack of software tools for identifying proteoforms from top‐down HetM‐MS/MS spectra. Many computational tools, like MSPathFinder [8], ProSightPD [9], TopPIC [10], and TopMG [11], have been developed for top‐down spectral identification through database searching. While these tools are efficient in identifying MS/MS spectra generated from single proteoforms, they are unable to identify multiple proteoforms from multiplexed spectra. Additionally, database search methods [12, 13, 14] for analyzing multiplexed bottom‐up DDA‐MS data are inefficient for processing multiplexed top‐down DDA‐MS data due to the complexity of top‐down mass spectra.

In this paper, we introduce TopMPI (TOP‐down mass spectrometry‐based Multiple Proteoform Identification), a new software tool designed to identify proteoform pairs from top‐down HetM‐MS/MS spectra via database searching. Experimental results demonstrate that TopMPI significantly improves proteoform identification from top‐down DDA‐MS data of complex biological samples compared to existing software tools, which focus solely on identifying single proteoforms from top‐down DDA mass spectra.

## 2 Materials and Methods

### 2.1 Reagents and Materials

Dithiothreitol (DTT), ammonium bicarbonate (ABC), Amicon Ultra‐0.5 filters (100 and 3 kDa) were purchased from Sigma‐Aldrich (St. Louis, MO). Phosphate buffered saline (PBS), protease inhibitor (EDTA free), formic acid (FA), acetonitrile (ACN, HPLC grade), isopropanol (IPA, HPLC grade), and water (HPLC grade) were obtained from Thermo Fisher Scientific (Waltham, MA, USA).

### 2.2 *E. coli* Sample Preparation

*E. coli* K12 MG1655 cells were pelleted by centrifugation at 5000 × *g* and 4°C for 5 min and then washed with 5 mL 1× PBS. The cell pellets were resuspended in 200 µL of 25 mM ABC buffer with the addition of 1× (v/v) EDTA‐free protease inhibitor. Then the cell pellets were lysed using 0.1 mm beads, which were mixed with the cell suspension and ABC buffer in a 1:1:2 (v/v) ratio, followed by bead beating for 3 min. After bead beating, the cell lysate was centrifuged at 12,000 × *g* and 4°C for 4 min to remove insoluble debris. Proteins were filtered using Amicon Ultra‐0.5 filters by centrifugation at 14,000 × *g* and 4°C for 20 min, retaining proteins with a molecular weight between 3 and 100 kDa. Next, 1 µL of 1 M DTT was added to the sample and incubated at 55°C for 45 min. The protein concentration was determined using the Pierce BCA Protein Assay Kit (Thermo Fisher Scientific, Waltham, MA, USA).

### 2.3 Top‐Down RPLC‐MS/MS Analysis

A total of 300 ng of *E. coli* protein was analyzed using an UltiMate 3000 reverse‐phase liquid chromatography (RPLC) system (Thermo Fisher Scientific, Waltham, MA, USA) equipped with a C2 column (100 µm i.d., 60 cm length, CoAnn, Richland, WA, USA) and coupled to an Orbitrap Fusion Lumos mass spectrometer (Thermo Fisher Scientific, San Jose, CA, USA). Mobile phase A was water with 0.1% FA. Mobile phase B was 60% ACN, 15% IPA, and 25% water with 0.1% FA. A 98‐min gradient (0–5 min 5%, 5–7 min for 5%–35%, 7–10 min for 35%–50%, 10–97 min for 50%–80%, 97–98 min for 80%–99%) was applied for proteoform separation with a flow rate of 400 nL/min.

MS1 scans were collected at a resolution of 240,000 (at 200 *m*/*z*) with 4 microscans, a maximum injection time of 200 ms, and a scan range of 720–1200 *m*/*z*. The top 6 precursors in each MS1 scan were selected for higher‐energy collisional dissociation (HCD) MS/MS analyses with the following settings: the precursor isolation window was 3 *m*/*z*, the normalized collision energy (NCE) was 30%, the automatic gain control (AGC) target was 10^6^, the maximum injection time was 500 ms, the microscan number was 1, the resolution was 60,000 (at 200 *m*/*z*), and the scan range was 400–2000 *m*/*z*.

### 2.4 Yeast Top‐Down MS Data

A public top‐down MS dataset of yeast proteins containing 62 capillary zone electrophoresis–tandem mass spectrometry (CZE‐MS/MS) replicate runs was downloaded from the PRIDE database (ID: PXD046651) [15]. In the experiments, an LPA‐coated capillary (50 µm i.d., 360 µm o.d., 1 m in length) was used for proteoform separation. MS/MS data were acquired using a Q Exactive HF mass spectrometer in the DDA mode. The MS and MS/MS spectra were collected at resolutions of 120,000 and 60,000 (at 200 *m*/*z*), respectively. The eight most intense precursor ions in each MS1 spectrum were isolated with a 2 *m*/*z* window to acquire HCD MS/MS spectra. The first and third replicates were used in the evaluation of TopMPI, while the second replicate was excluded because of its relatively low number of proteoform identifications. The first and third replicates contained 7632 and 9480 MS/MS spectra, respectively.

### 2.5 Top‐Down Mass Spectral Preprocessing

Top‐down MS raw data files were converted to centroided mzML files using msconvert [16], and then the mzML files were analyzed by TopFD [17] (version 1.7.6 and Table S1 for parameter settings) for spectral deconvolution and feature detection. A proteoform feature reported by TopFD includes all peaks of the proteoform with various isotopic compositions, charge states, and retention times observed in MS1 spectra. For an MS/MS spectrum and a proteoform, the *precursor intensity* of the proteoform is defined as the total intensity of all the peaks that are included in the proteoform feature and located within the isolation window in the corresponding MS1 spectrum. For each MS/MS spectrum, we selected the two proteoforms with the highest precursor intensities and assigned them as the spectrum's precursor proteoforms.

### 2.6 Primary Precursor Selection

**FIGURE 1:**
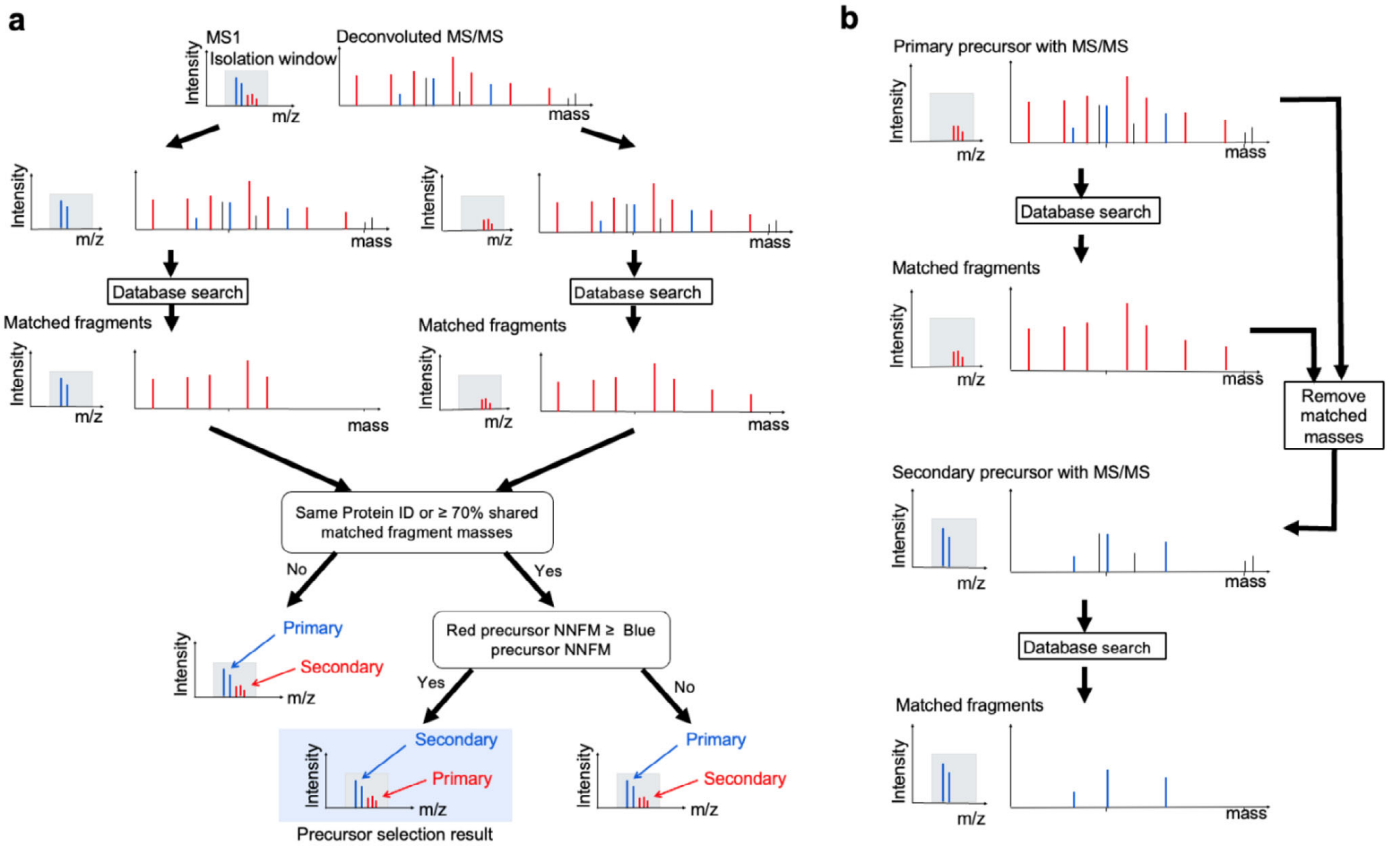
The overview of TopMPI. (a) Primary precursor selection for a multiplexed MS/MS spectrum with two precursors (blue and red) in the MS1 spectrum, along with their corresponding fragment masses (blue and red) and noise fragments (black). The red and blue precursors, paired with all fragment masses, are searched separately against a protein sequence database, resulting in a correct identification with no mass shifts for the red precursor and an incorrect identification with a mass shift for the blue precursor. The red precursor is selected as the primary precursor because the two PrSMs share more than 70% of matched fragment masses and the NNMFM of the red precursor is higher than that of the blue precursor. (b) Two‐round database search strategy. The primary precursor (red) and all fragment masses in the spectrum are initially searched against the protein sequence database for proteoform identification. Once a match is found, the matched fragment masses (red) are removed. The secondary precursor (blue) and the remaining fragment masses are then searched against the protein sequence database for proteoform identification.

TopMPI is designed to identify two proteoforms from two distinct proteins from a top‐down HetM‐MS/MS spectrum with two precursors. Its primary function is to determine the order of the two precursors for database search‐based proteoform identification (Figure 1a). Precursor selection errors (PSEs) are common in multiplexed top‐down mass spectral identification, where the precursor with the highest intensity of an MS/MS spectrum is incorrectly paired with fragment masses from a different precursor of the MS/MS spectrum, resulting in an erroneous proteoform identification. TopMPI mitigates PSEs by evaluating both precursors and dynamically selecting the order of the two precursors before performing database search.

For an MS/MS spectrum *S* with two precursors *F*_1_ and *F*_2_, where *F*_1_ has the highest and *F*_2_ has the second highest precursor intensities, the spectrum *S* is classified as a multiplexed one if the precursor intensity ratio between *F*_2_ and *F*_1_ exceeds a predefined threshold 𝛼 (𝛼 = 20% in the experiments, see Section 3.2). Otherwise, the MS/MS spectrum is treated as a non‐multiplexed one, and only *F*_1_ is assigned to the MS/MS spectrum as its precursor for proteoform identification.

For a multiplexed spectrum with two precursors, two rounds of database searches are conducted to identify two proteoforms. The precursor used in the first round of database search is referred to as the primary precursor, while the other is designated as the secondary precursor. Notably, the primary precursor may have a lower intensity than the secondary precursor.

For spectrum *S*, the primary precursor is determined as follows. Define *S*_1_ as the precursor‐fragment paired spectrum (PFPS) formed by combining precursor *F*_1_ and all fragment masses in *S*. Likewise, define *S*_2_ as the PFPS generated by combining precursor *F*_2_ and all fragment masses in *S*. The two PFPSs are searched against the corresponding protein sequence database for proteoform identification using TopPIC (version 1.7.6) without filtering of reported proteoform‐spectrum matches (PrSMs) (Figure 1a). If TopPIC reports a PrSM from only one PFPS and fails to identify any PrSMs from the other, the precursor corresponding to the reported PrSM is selected as the primary precursor. If a PrSM is reported for each of the two PFPSs, we will first check whether there are PSEs in the two reported PrSMs.

TopMPI uses two methods to find PrSMs with possible PSEs. Suppose that precursor proteoform *F*_1_ is from a protein *P*_1_ and precursor proteoform *F*_2_ is from another protein *P*_2_. First, when the fragment masses of *F*_1_ are incorrectly paired with precursor *F*_2_ in the identification of the PrSM of *S*_2_, the PrSM of *S*_2_ is often matched to a proteoform of *P*_1_. That is, both *S*_1_ and *S*_2_ are matched to proteoforms of the same protein *P*_1_. Therefore, if the two PrSMs reported from *S*_1_ and *S*_2_ are matched to the same protein, then one of the two PrSMs may have a PSE and we report spectrum *S* as an error‐prone spectrum. Second, a high number of shared matched fragment masses between the two PrSMs is also indicative of a PSE because two proteoforms from two distinct proteins seldom share many matched fragment masses. Let *M*_1_ and *M*_2_ represent the sets of matched experimental fragment masses in the two PrSMs reported by database search, respectively. The ratio of shared matched fragment masses is computed as $\frac{|M_{1}\cap M_{2}|}{\min\{|M_{1}\left|,\right|M_{2}|\}}$, where |.| is the size of a set. If the ratio ≥ β, where β is the user specified parameter (β=0.7 in the experiments, see Section 3.2), we report spectrum *S* as an error‐prone spectrum.

If spectrum *S* is not error‐prone, the order of *F*_1_ and *F*_2_ in database search does not significantly affect proteoform identifications and *F*_1_ can be arbitrarily chosen as the primary precursor. However, if spectrum *S* is error‐prone, the primary precursor must be carefully selected to mitigate PSEs. Typically, in such cases, only one of the two PrSMs of *S*_1_ and *S*_2_ contains a PSE, while the other does not. That is, one PrSM is correct and the other is incorrect (Figure 1a). If the precursor of the correct PrSM is selected as the primary one, the PSE can be avoided in database search‐based proteoform identification (Figure 1b). In TopMPI, the precursor corresponding to the PrSM with the higher number of normalized matched fragment masses (NNMFM) is selected as the primary precursor, as the PrSM with a PSE typically have fewer matched fragment masses than the other PrSM without a PSE. We consider two factors in the normalization of the number of matched fragment masses: (1) the number of unknown mass shifts in the PrSM, and (2) the abundance of the precursor. For a PrSM with unknown mass shifts, a penalty *δ* (*δ* = 5 in the experiments, see Section 3.2) is added for each unknown mass shift. Because the precursor with the second highest intensity in general tends to generate less fragment masses than the precursor with the highest intensity, another penalty *𝛾* (*𝛾* = 4 in the experiments, see Section 3.2) is applied to the PrSM of the precursor with the second highest intensity. For a PrSM with *x* matched fragment masses and *y* unknown mass shifts, the NNMFM is computed as *x*‐*yδ*‐z*𝛾*, where *z* = 0 for the precursor with the highest intensity and *z* = 1 for the precursor with the second highest intensity.

### 2.7 Proteoform Identification by Multiplexed Top‐Down DDA Mass Spectra

TopMPI employs a method similar to CharmeST [14] to identify two proteoforms from multiplexed top‐down DDA mass spectra (Figure 1b). For an MS/MS spectrum *S* with a primary precursor and a secondary precursor, a two‐round database search is conducted to identify two proteoforms. In the first round, the primary precursor and the fragment masses in spectrum *S* are searched against a protein sequence database using TopPIC (version 1.7.6). If a PrSM is reported, the matched fragment masses of the PrSM are removed from *S*. In the second round, the secondary precursor and the remaining fragment masses in *S* are searched against the same protein sequence database using TopPIC. If the two PrSMs identified in the two rounds are matched to the same protein, then only the PrSM with the lower *E* value is kept. The PrSMs identified in the first and second rounds are filtered separately using an E‐value or spectrum‐level false discovery rate (FDR) cutoff. The filtered PrSMs from both rounds are then merged and clustered into proteoform groups. Two PrSMs are grouped together if their precursors are from the same proteoform feature reported by TopFD [17] or if they are matched to the same protein and their precursor mass difference is less than 1.2 Da. Finally, the resulting proteoforms are filtered using an *E*‐value or proteoform‐level FDR cutoff.

## 3 Results

### 3.1 Generation of Pseudo Multiplexed MS/MS Spectra

To assess the performance of TopMPI in identifying multiplexed spectra, we generated evaluation datasets of pseudo‐multiplexed MS/MS spectra from the top‐down DDA‐MS dataset containing 10,320 MS/MS spectra generated from *E. coli* K12 MG1655 cells (see Section 2). After spectral deconvolution and feature detection, TopPIC (version 1.7.6) was used to search the MS/MS spectra against the UniProt *E. coli* K12 protein database (version September 7, 2023; 4530 entries) and identified 1935 PrSMs using an E‐value cutoff of 0.01 (parameter settings in Table S2). Since some of the identified MS/MS spectra were multiplexed, the spectra were filtered based on the precursor intensities observed in their isolation windows in MS1 spectra. If the intensity of the highest intensity precursor was less than 85% of the total intensity of all precursors, the MS/MS spectrum was excluded. The remaining 566 spectra were treated as non‐multiplexed spectra. Note that the method used here for selecting non‐multiplexed spectra differs from the method in TopMPI. A non‐multiplexed spectrum was classified as a zero‐shift spectrum if its proteoform contained no unknown mass shifts and as a one‐shift spectrum if its proteoform contained one unknown mass shift. Among the 566 spectra, 319 were zero‐shift spectra, and 247 were one‐shift spectra.

First, we generated an evaluation dataset of pseudo‐multiplexed MS/MS spectra using the 566 non‐multiplexed MS/MS spectra. A pair of spectra from the 566 non‐multiplexed spectra was considered a matched pair if it met three criteria: (1) the charge states of their precursors were different, (2) the difference between the average *m*/*z* values of their precursors was no more than 1.5 *m*/*z*, and (3) their proteoform identifications were from two different proteins. A total of 1353 matched spectrum pairs were identified, and a pseudo‐multiplexed MS/MS spectrum was generated for each pair by combining the fragment masses of the two spectra. The spectrum with more matched fragment masses in its proteoform identification was designated as the base spectrum in the multiplexed spectrum, while the other spectrum was designated as the additive spectrum. The protein identifications of the base and additive spectra are referred to as the base protein and additive protein, respectively. We also assigned a higher intensity to the precursor of the base spectrum than the precursor of the additive spectrum. The dataset is referred to as the simple paired multiplexed spectra (SPMS) dataset.

Second, we generated evaluation datasets of pseudo‐multiplexed MS/MS spectra by controlling the number of fragment masses associated with each of the two precursors in the multiplexed MS/MS spectrum. Similar to the SPMS data, a non‐multiplexed experimental spectrum was selected as the base spectrum, and another non‐multiplexed spectrum was chosen as the additive spectrum. A spectrum was considered a valid additive spectrum for a given base spectrum if it satisfied four criteria: (1) the precursors of the base and additive spectra were of two different charge states, (2) the distance between the average *m/z* values of the precursors of the additive and base spectra was no greater than 20 *m*/*z*, (3) the proteoform identifications of the base and additive spectra were from two different proteins, and (4) after removing from the additive spectrum the fragment masses that are randomly matched to the b‐ or y‐ions of the proteoform of the base spectrum, the number of remaining fragment masses in the additive spectrum was at least twice that of the base spectrum.

Among the 566 non‐multiplexed spectra, 338 had both a valid zero‐shift additive spectrum and a valid one‐shift additive spectrum. Of these 338 base spectra, 190 were zero‐shift spectra, and 148 were one‐shift spectra. The 338 base spectra and their corresponding zero‐shift additive spectra were used to generate 338 spectral pairs. The spectral pairs in which the base spectrum was a zero‐shift or one‐shift spectrum were referred to as Base0‐Add0 and Base1‐Add0 pairs, respectively. Similarly, the 338 base spectra and their corresponding one‐shift additive spectra were used to generate an additional 338 spectral pairs, and those with zero‐shift and one‐shift base spectra were referred to as Base0‐Add1 and Base1‐Add1 pairs, respectively. In total, 676 spectral pairs were generated. For each pair, we assigned a higher intensity to the precursor of the base spectrum than the precursor of the additive spectrum.

The 676 spectral pairs were used to generate 11 pseudo‐multiplexed spectrum datasets, each corresponding to an additive‐base ratio (A‐B ratio) of 0%, 20%, 40%, …, 200%. The dataset with an A‐B ratio contained 676 spectra, each containing all fragment masses from the base spectrum and a randomly selected subset of fragment masses from the additive spectrum, such that the ratio between the number of additive fragment masses and that of base fragment masses approximately equals to the A‐B ratio. The datasets are referred to as ratio paired multiplexed spectra (RPMS) datasets.

### 3.2 Selection of Default Parameters

The first replicate of the top‐down MS yeast dataset containing 7632 MS/MS spectra was used to investigate how to set the parameter 𝛼 for filtering non‐multiplexed spectra (see Section 2). Following data preprocessing (see Section 2), TopPIC (version 1.7.6; parameter settings in Table S3) was used to search the MS/MS spectra against the UniProt yeast protein database (version March 3, 2023; 6727 entries) concatenated with a decoy database of equal size. In TopPIC, for each MS/MS spectrum, only the precursor with the highest intensity was paired with fragment masses for proteoform identification. Once the first PrSM was reported, the matched fragment masses corresponding to the identified proteoform were removed, and a new MS/MS spectrum was generated using the precursor with the second highest intensity and the remaining unmatched fragment masses. This newly generated spectrum was then searched against the same target‐decoy database using TopPIC with the same parameter settings. The PrSMs reported from the two precursors were filtered separately using a 1% spectrum‐level FDR. In addition, if the proteoform identifications from the two precursors were from the same protein, the proteoform identification from the second precursor was removed.

A total of 4589 and 890 PrSMs were identified from the first and second precursors, respectively, with 615 spectra with proteoform identifications from both precursors. The intensity ratio between the second and first precursors was calculated for each of the 615 spectra (Figure S1), and most of the spectra had a ratio that exceeded 20%. Based on this observation, the parameter 𝛼 for filtering non‐multiplexed spectra was set to 0.2. Consequently, in the experiments, a spectrum was classified as multiplexed only if the intensity ratio between the second and first precursors was at least 20%.

We also used the first replicate of the yeast dataset to study the setting of the parameter β, which uses the ratio of shared matched fragment masses between the PrSMs reported from the first and second precursors to identify error‐prone spectra (see Section 2). After filtering out MS/MS spectra with 𝛼 = 20%, 3721 spectra remained. For each of the 3721 spectra, we paired the fragment masses with the two precursors with the highest intensities separately and searched the two resulting spectra against the UniProt yeast proteome database (version March 3, 2023, 6727 entries) concatenated with a shuffled decoy database of the same size using TopPIC (parameter settings in Table S3) separately. Among the 3721 multiplexed spectra, proteoform pair identifications were reported for 622 spectra using a spectrum‐level FDR cutoff of 0.01. The distribution of the ratios of shared matched fragment masses between the PrSM pairs from the 622 spectra (Figure S2) indicated that a cutoff value of 0.7 for *β* effectively distinguished pairs with high overlap from those with low overlap. Therefore, the default value of *β* was set to 0.7.

We further evaluated the error rates of TopMPI under different settings for *δ* and *γ* (see Section 2) using the RPMS dataset with an A‐B ratio of 100%. The mass spectra were searched against the UniProt *E. coli* K12 proteome database (version September 7, 2023; 4530 entries) concatenated with a shuffled decoy database of the same size using TopMPI (parameter settings in Table S4). The identified PrSMs were filtered using a 1% spectrum‐level FDR. TopMPI reported two types of incorrect spectral identifications. The first type is a PSE (see Section 2). The second type, referred to as a random matching error (RME), occurred when the precursor of the base or additive spectrum was matched to a protein that was neither the base protein nor the additive protein. A comparison of the performance of TopMPI under various settings for *δ* and *γ* showed that the lowest PSE rate was achieved when *δ* = 0, 1, …, 8 and *γ* = 3, 4, …, 8 (Figure S3). We selected *δ* = 5 and *γ* = 4 as the default settings for TopMPI. Additionally, the settings of *δ* and *γ* did not significantly impact the RME rates.

### 3.3 Comparison of TopPIC and TopMPI

**FIGURE 2:**
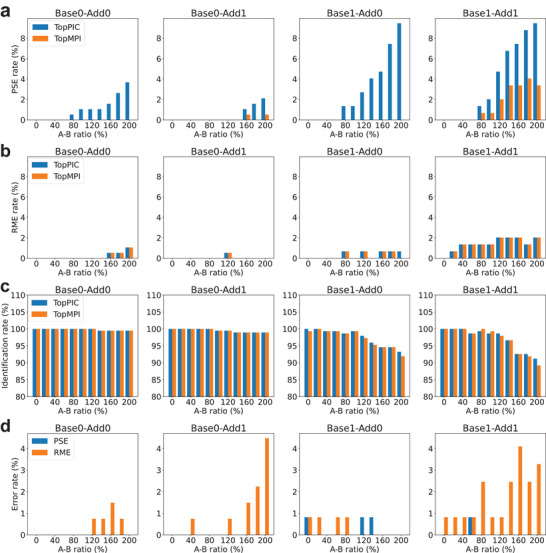
Error rates and identification rates of PrSMs reported by TopPIC and TopMPI at a 1% spectrum‐level FDR across four types of pseudo‐multiplexed spectra with varying A‐B ratios (0%, 20%, …, 200%) in the RPMS datasets. (a) PSEs of PrSMs from base precursors. (b) RMEs of PrSMs from base precursors. (c) Identification rates for base precursors. (d) PSEs and RMEs of PrSMs from additive precursors reported by TopMPI.

We first compared the accuracy of proteoform identifications of TopPIC (version 1.7.6) and TopMPI using the 11 RPMS datasets. The mass spectra were searched separately against the target‐decoy UniProt *E. coli* K12 proteome database using TopPIC and TopMPI (parameter settings provided in Tables S3 and S5). The identified PrSMs were filtered using a 1% spectrum‐level FDR. As the A‐B ratio increased, the error rates of spectral identifications reported by TopPIC also increased across the four types of spectrum pairs (Figure 2a,b). The majority of errors reported by TopPIC were PSEs, with higher error rates when the proteoform of the base spectrum contained unknown mass shifts. For base precursors, TopMPI significantly reduced PSEs in spectral identifications (Figure 2a) while reporting a similar number of RMEs (Figure 2b) compared to TopPIC. However, the identification sensitivity of TopMPI was slightly lower than TopPIC (Figure 2c). One possible explanation for this decrease in sensitivity is that TopMPI removes some identifications to correct PSEs. When a base precursor is incorrectly matched to the additive protein, TopMPI swaps the order of the base and additive precursors in database search to correct the error, thereby also removing the incorrect identification of the base precursor. For additive precursors, TopMPI reported slightly higher rates of PSEs and RMEs compared to base precursors (Figure 2a,b,d).

**FIGURE 3:**
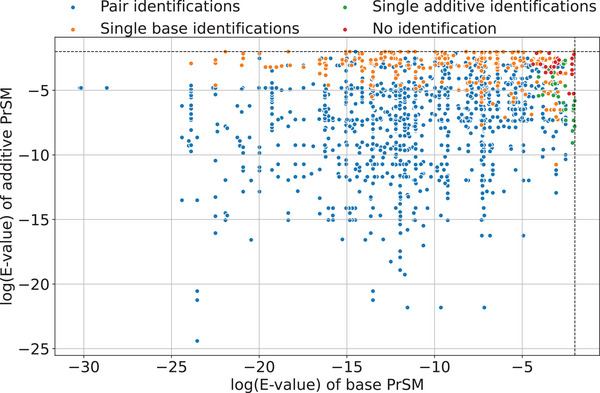
Distributions of log E‐values (base 10) for the PrSMs of the base and additive non‐multiplexed spectra in the 1353 pseudo‐multiplexed spectra in the SPMS dataset. The *E* values of the two PrSMs for the two non‐multiplexed spectra in each pseudo‐multiplexed spectrum were reported by TopPIC. TopMPI reported 1055 proteoform pair identifications (blue), 244 single proteoform identifications for base spectra (orange), 28 single proteoform identifications for additive spectra (green), and missed identifications for 26 spectra (red).

Second, we compared the number of proteoform identifications of TopPIC (version 1.7.6) and TopMPI using the SPMS dataset with 1353 pseudo‐multiplexed spectra. All the spectra were searched against the UniProt *E. coli* K12 proteome database (version September 7, 2023; 4530 entries) using TopPIC and TopMPI (parameter settings provided in Tables S2 and S6). PrSMs reported by the two tools were filtered with an *E*‐value cutoff of 0.01. TopPIC identified 1299 PrSMs, of which 6 (0.5%) were incorrect. TopMPI reported PrSM pairs for 1055 spectra and single PrSMs for 272 spectra, of which 244 identifications were from the base spectra and 28 from the additive spectra (Figure 3). TopMPI identified a total of 2382 PrSMs, representing an 83.4% increase in PrSM identifications compared to TopPIC. Among the 2382 PrSMs, 2374 (99.7%) were correct, 7 (0.3%) contained PSEs, and 1 (0.1%) had RMEs. The overall error rate of TopMPI was comparable to that of TopPIC. The *E*‐value distribution of the original PrSMs used to construct the pseudo‐multiplexed spectra revealed that TopMPI tends to miss PrSMs with low‐confidence identifications in the original non‐multiplexed spectra. Because the additive spectra contained fewer matched fragments than the base spectra, more missing identifications were observed for additive spectra compared to base spectra.

**FIGURE 4:**
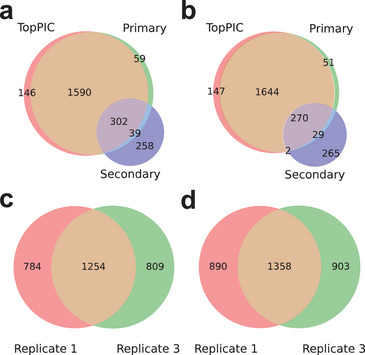
Comparison of proteoform identifications reported by TopPIC and TopMPI from replicates 1 and 3 of the yeast dataset. Venn diagrams showing proteoform identifications reported by TopPIC and by TopMPI from primary and secondary precursors in replicate 1 (a) and replicate 3 (b). Venn diagrams of proteoform identifications reported from replicates 1 and 3 by TopPIC (c) and TopMPI (d). The ratios between the overlapping identifications and those reported from replicate 1 are 61.5% and 60.4% for TopPIC and TopMPI, respectively.

Third, we compared spectral identifications reported by TopMPI and TopPIC on the first and third replicates of the top‐down CZE‐MS/MS dataset of yeast proteins (Pride ID: PXD046651) containing 7632 and 9480 MS/MS spectra, respectively (see Section 2). Following data preprocessing, TopPIC (version 1.7.6) and TopMPI were used to search the MS/MS spectra against the UniProt yeast proteome database (version March 3, 2023; 6727 entries) with parameter settings detailed in Tables S7 and S8. In both methods, to reduce PrSM identifications with unknown mass shifts, the minimum and maximum mass shift values observed in a PrSM identification were set to ‐50 and 200 Da, respectively. In addition, PrSM and proteoform identifications were filtered using a 1% FDR. For replicate 1, TopPIC identified 4550 PrSMs and 2038 proteoforms, while TopMPI increased PrSM identifications by 22.0% (5549 PrSMs) and proteoform identifications by 10.3% (2248 proteoforms) (Figure 4a). Among the TopMPI identifications, 1649 proteoforms were from primary precursors only, and 258 proteoforms were from secondary precursors only. The primary and secondary searches shared 341 proteoforms, which was 17.1% of the total proteoform identifications from the primary search and 56.9% of the total proteoform identifications from the secondary search. An example proteoform pair identification reported by TopMPI is given in Figure S4 and Table S9. TopMPI identified 356 proteoforms missed by TopPIC, of which 59 (16.6%) were from primary identifications only, 258 (72.5%) from secondary identifications only, whereas the remaining 39 (11.0%) were reported by both primary and secondary identifications. Similarly, for replicate 3, TopPIC identified 4,439 PrSMs and 2063 proteoforms, while TopMPI increased PrSM identifications by 22.1% (5420 PrSMs) and proteoform identifications by 9.6% (2261 proteoforms) (Figure 4b). Of the TopMPI identifications, 1695 proteoforms were from primary precursors only, 267 proteoforms were from secondary precursors only, and 299 proteoforms were shared by primary and secondary precursor identifications. Furthermore, TopMPI demonstrated comparable reproducibility (60.4%) of proteoform identifications across the two replicates compared to TopPIC (61.5%) (Figure 4c,d).

We further investigated the mass shifts in the 1649 proteoforms reported from the primary precursors only and the 258 proteoforms reported from the secondary precursors only by TopMPI from the first replicate (Figures S5 and S6). The 1659 proteoforms of the primary precursors contained 283 mass shifts, of which 94 (33.2%) were matched to common mass shifts (Table S10). And the 258 proteoforms of the secondary precursors contained 66 mass shifts, of which 15 (22.7%) were matched to common ones (Table S10). A total of 19.8% (15) of the proteoforms of secondary precursors contained an uncommon mass shift, which was higher than 11.4% (189) of the proteoforms of primary precursors with an uncommon mass shift. Because secondary precursors typically have lower abundances than the primary precursors and low abundance precursors tend to have more errors in spectral deconvolution than high abundance ones, the secondary precursors might have a higher error rate in deconvoluted masses than the primary precursors. This might be a reason for the higher percentage of uncommon mass shifts in the proteoform identifications of the secondary precursors. We also studied the mass shifts in the 356 proteoforms identified by TopMPI but missed by TopPIC. Of the 356 proteoforms, only 79 (22.2%) contained mass shifts, of which 16 were matched to common ones: 2 disulfide bonds, 7 first isotopic peaks, 1 methylation, 1 oxidation, 1 sodium cation, 1 acetylation, 1 iron (III) cation, 2 phosphorylation sites (Figure S7), and the remaining 63 (17.0%) contained uncommon mass shifts.

### 3.4 Comparison of MSPathFinder and TopMPI

We compared proteoform identifications reported by MSPathFinder [8] and TopMPI on the first replicate of the yeast dataset. ProMex (version 1.1.8305 and parameter settings in Table S11) was used for spectral deconvolution and MSPathFinder (version 1.1.8305 and parameter settings in Table S12) was employed to search the deconvoluted MS/MS spectra against the UniProt yeast proteome database (version March 3, 2023; 6727 entries). MSPathFinder identified 1,194 proteoforms with a 1% proteoform FDR, which was less than the proteoform identifications reported by TopMPI (2,248). And TopMPI identified 1307 proteoforms missed by MSPathFinder (Figure S8).

### 3.5 Discussion and Conclusions

We developed TopMPI, a software tool for identifying proteoform pairs from top‐down HetM‐MS/MS spectra. TopMPI consists of two main functions. The first is to determine the primary precursor within the isolation window, which is expected to generate most of the fragment ions in the MS/MS spectrum. The second function employs a greedy approach, first identifying a PrSM using the primary precursor, then removing the matched fragment masses, and subsequently using the secondary precursor and the remaining fragment masses to identify another PrSM.

TopMPI reduces PSEs in PrSMs reported from multiplexed MS/MS spectra compared to existing tools designed for non‐multiplexed spectra identification, such as TopPIC [10]. PSEs are commonly observed when multiplexed spectra are incorrectly treated as non‐multiplexed ones in spectral identification. The precursor selection function in TopMPI effectively corrects many of the PSEs observed in proteoform identifications reported by TopPIC.

Beyond reducing PSEs, TopMPI also increases proteoform identifications compared to tools designed for non‐multiplexed spectra identification. In top‐down MS analysis of complex samples, it is common that multiple proteoforms are coeluted within the same isolation window. These multiplexed spectra are often treated as non‐multiplexed, where only the precursor with the highest signal intensity is selected for spectral identification. This approach fails to identify proteoforms whose precursor intensities are not the highest within the isolation window. TopMPI enables the identification of these proteoforms, thereby increasing the sensitivity of proteoform identification.

Spectral deconvolution may report incorrect precursor masses for top‐down multiplexed MS/MS spectra, and these incorrect precursors may cause incorrect proteoform identifications in TopMPI. Two methods can be used to mitigate the problem of incorrect precursors. First, confidence scores of precursors, such as ECScores reported by TopFD [17], can be used to filter out low confidence ones. Second, proteoforms reported by TopMPI can be filtered to keep only those without any PTMs or with mass shifts corresponding to a user‐specified list of PTMs.

TopMPI still has several limitations. First, it determines the primary precursor based solely on the NNMFMs of PrSMs of precursors, which is insufficient to correct all PSEs. The correlation between extracted ion chromatograms (XICs) of precursor and fragment ions has been effectively used to assign fragment ions to their corresponding precursor ions in the analysis of multiplexed bottom‐up and top‐down mass spectra in DIA‐MS [4, 18, 19]. A complex multiplexed spectrum can be demultiplexed by assigning each fragment ion to the precursor ion with the highest XIC correlation. In top‐down DDA‐MS, this approach can help reduce PSEs when multiple MS/MS spectra are acquired for the same precursor. Second, TopMPI employs a greedy approach for spectral identification, where the *E* value of the primary precursor identification tends to be less significant due to the presence of fragment masses from the secondary precursor in the spectrum. This can lower the sensitivity of spectral identification. An alternative approach is to directly match a multiplexed spectrum to a combination of two proteoforms, which may help address this issue and enhance spectral identification sensitivity. Third, TopMPI is currently unable to identify multiple proteoforms from HomM‐MS/MS spectra, even though such spectra are commonly encountered when we analyze proteins with many potential PTM sites. Identification of such spectra is particularly challenging because co‐fragmented proteoforms originating from the same protein are often highly similar and share many fragment ions.

## Conflicts of Interest

X.L. has a project contract with Bioinformatics Solutions Inc., a company that develops software for MS data processing.

## Language Polishing

The authors used ChatGPT to enhance the language and readability during the preparation of this paper. After utilizing ChatGPT, the authors reviewed and edited the content and take full responsibility for the final version of the paper.

## Supporting information




**Supporting File 1**: pmic70020‐sup‐0001‐SuppMat.docx

## Data Availability

The source code of TopMPI is available at https://github.com/todanielwang/TopMPI. The *E. coli* and the pseudo‐multiplexed spectra datasets (labeled as SPMS and RPMS datasets) are publicly accessible on MassIVE (ID: MSV000097090).
