# Supplementary material for: Proteoform Identification Using Multiplexed Top‐Down Mass Spectra
Source: Proteomics. 2025 Jul 30;25(24):67–76. doi: 10.1002/pmic.70020 (PMC12716115; doi:10.1002/pmic.70020)
Supplement: Supplementary file 1 — Supporting File 1: pmic70020‐sup‐0001‐SuppMat.docx [file PMIC-25--s001.docx]

**Proteoform identification using multiplexed top-down mass spectra**

**(Supplemental materials)**

Zhige Wang^1^, Xingzhao Xiong^2^, and Xiaowen Liu^2^

^1^Department of Computer Science, Tulane University, New Orleans, Louisiana, 70112, United States

^2^Deming Department of Medicine, Tulane University, New Orleans, Louisiana, 70112, United States

**Tables**

**Table S1**: Parameter settings of TopFD

| Input Parameter | Value |
| --- | --- |
| Maximum charge | 30 |
| Maximum mass | 70,000 Da |
| m/z error tolerance of spectral peaks | 0.02 m/z |
| MS1 signal-to-noise ratio | 3 |
| MS/MS signal-to-noise ratio | 1 |
| Use MSDeconv score | False |
| ECScore cutoff | 0.5 |
| Number of MS1 scans to detect a feature | 3 |
| Use noise levels in single scans to filter | False |
| Disable final filtering of envelopes | False |
| Disable additional feature search | False |

**Table S2**: Parameter settings of TopPIC using E-value cutoffs

| Input Parameter | Value |
| --- | --- |
| Fixed modification | None |
| Allowed N-terminal forms | None, NME, NME_ACETYLATION, M_ACETYLATION |
| Maximum number of mass shift | 1 |
| Minimum value of a mass shift | -500 Da |
| Maximum value of a mass shift | 500 Da |
| Use a shuffled decoy database | False |
| Mass error tolerance | 10 ppm |
| Proteoform error tolerance | 1.2 Da |
| Spectrum level cutoff type | E-value |
| Spectrum level cutoff value | 0.01 |
| Proteoform level cutoff type | E-value |
| Proteoform level cutoff value | 0.01 |
| Use TopFD Features | True |

**Table S3**: Parameter settings of TopPIC using FDR cutoffs

| Input Parameter | Value |
| --- | --- |
| Fixed modification | None |
| Allowed N-terminal forms | None, NME, NME_ACETYLATION, M_ACETYLATION |
| Maximum number of mass shift | 1 |
| Minimum value of a mass shift | -500 Da |
| Maximum value of a mass shift | 500 Da |
| Use a shuffled decoy database | True |
| Mass error tolerance | 10 ppm |
| Proteoform error tolerance | 1.2 Da |
| Spectrum level cutoff type | FDR |
| Spectrum level cutoff value | 0.01 |
| Proteoform level cutoff type | FDR |
| Proteoform level cutoff value | 0.01 |
| Use TopFD Features | True |

**Table S4**: Parameter settings of TopMPI for selecting the settings for *δ* and *γ*

| Input Parameter | Value |
| --- | --- |
| Fixed modification | None |
| Allowed N-terminal forms | None, NME, NME_ACETYLATION, M_ACETYLATION |
| Maximum number of mass shift | 1 |
| Minimum value of a mass shift | -500 Da |
| Maximum value of a mass shift | 500 Da |
| Use a shuffled decoy database | True |
| Mass error tolerance | 10 ppm |
| Proteoform error tolerance | 1.2 Da |
| TopPIC spectrum level cutoff type | E-value |
| TopPIC spectrum level cutoff value | 10,000 |
| TopMPI spectrum level cutoff type | FDR |
| TopMPI spectrum level cutoff value | 0.01 |
| TopMPI use TopFD Features | Yes |
| TopMPI proteoform level cutoff type | FDR |
| TopMPI proteoform level cutoff type | 0.01 |
| *α* | 0.2 |
| *β* | 0.7 |
| *δ* | Various settings |
| *γ* | Various settings |
| Feature ECScore cutoff | 0 |

**Table S5**: Parameter settings of TopMPI with FDR cutoffs

| Input Parameter | Value |
| --- | --- |
| Fixed modification | None |
| Allowed N-terminal forms | None, NME, NME_ACETYLATION, M_ACETYLATION |
| Maximum number of mass shift | 1 |
| Minimum value of a mass shift | -500 Da |
| Maximum value of a mass shift | 500 Da |
| Use a shuffled decoy database | True |
| Mass error tolerance | 10 ppm |
| Proteoform error tolerance | 1.2 Da |
| TopPIC spectrum level cutoff type | E-value |
| TopPIC spectrum level cutoff value | 10,000 |
| TopMPI spectrum level cutoff type | FDR |
| TopMPI spectrum level cutoff value | 0.01 |
| TopMPI use TopFD Features | Yes |
| TopMPI proteoform level cutoff type | FDR |
| TopMPI proteoform level cutoff type | 0.01 |
| *α* | 0.2 |
| *β* | 0.7 |
| *δ* | 5 |
| *γ* | 4 |
| Feature ECScore cutoff | 0 |

**Table S6**: Parameter settings of TopMPI with E-value cutoffs

| Input Parameter | Value |
| --- | --- |
| Fixed modification | None |
| Allowed N-terminal forms | None, NME, NME_ACETYLATION, M_ACETYLATION |
| Maximum number of mass shift | 1 |
| Minimum value of a mass shift | -500 Da |
| Maximum value of a mass shift | 500 Da |
| Use a shuffled decoy database | True |
| Mass error tolerance | 10 ppm |
| Proteoform error tolerance | 1.2 Da |
| TopPIC spectrum level cutoff type | E-value |
| TopPIC spectrum level cutoff value | 10,000 |
| TopMPI spectrum level cutoff type | E-value |
| TopMPI spectrum level cutoff value | 0.01 |
| TopMPI use TopFD Features | Yes |
| TopMPI proteoform level cutoff type | E-value |
| TopMPI proteoform level cutoff type | 0.01 |
| *α* | 0.2 |
| *β* | 0.7 |
| *δ* | 5 |
| *γ* | 4 |
| Feature ECScore cutoff | 0 |

**Table S7**: Parameter settings of TopPIC for analyzing the yeast dataset

| Input Parameter | Value |
| --- | --- |
| Fixed modification | None |
| Allowed N-terminal forms | None, NME, NME_ACETYLATION, M_ACETYLATION |
| Maximum number of mass shift | 1 |
| Minimum value of a mass shift | -50 Da |
| Maximum value of a mass shift | 200 Da |
| Use a shuffled decoy database | True |
| Mass error tolerance | 10 ppm |
| Proteoform error tolerance | 1.2 Da |
| Spectrum level cutoff type | FDR |
| Spectrum level cutoff value | 0.01 |
| Proteoform level cutoff type | FDR |
| Proteoform level cutoff value | 0.01 |
| Use TopFD Features | True |

**Table S8**: Parameter settings of TopMPI for analyzing the yeast dataset

| Input Parameter | Value |
| --- | --- |
| Fixed modification | None |
| Allowed N-terminal forms | None, NME, NME_ACETYLATION, M_ACETYLATION |
| Maximum number of mass shift | 1 |
| Minimum value of a mass shift | -50 Da |
| Maximum value of a mass shift | 200 Da |
| Use a shuffled decoy database | True |
| Mass error tolerance | 10 ppm |
| Proteoform error tolerance | 1.2 Da |
| TopPIC spectrum level cutoff type | E-value |
| TopPIC spectrum level cutoff value | 10,000 |
| TopMPI spectrum level cutoff type | FDR |
| TopMPI spectrum level cutoff value | 0.01 |
| TopMPI use TopFD Features | Yes |
| TopMPI proteoform level cutoff type | FDR |
| TopMPI spectrum level cutoff type | 0.01 |
| *α* | 0.2 |
| *β* | 0.7 |
| *δ* | 5 |
| *γ* | 4 |
| Feature ECScore cutoff | 0 |

**Table S9**: Two proteoforms identified from scan 2450 in the first replicate of the yeast dataset

| **Precursor** | | | | **Proteoform** | | |
| --- | --- | --- | --- | --- | --- | --- |
|  | **Avg m/z** | **Charge** | **Mono mass** | **Accession** | **AAs** | **Sequence** |
| Primary | 833.14 | 11 | 9147.66 | sp\|P00925\|ENO2_YEAST | 354 - 437 | SESIKAAQDSFAANWGVMVSHRSGETEDTFIADLVVGLRTGQIKTGAPARSERLAKLNQLLRIEEELGDKAVYAGENFHHGDKL |
| Secondary | 833.59 | 5 | 4160.30 | sp\|P00950\|PMG1_YEAST | 28 - 65 | LSAKGQQEAARAGELLKEKKVYPDVLYTSKLSRAIQTA |

**Table S10**: List of common mass shifts used in the mass shift analysis

| **Modification** | **Monoisotopic Mass** |
| --- | --- |
| Disulfide bond | -2.02 Da |
| First Isotopic Peak | +1.003 Da |
| Methylation | +14.02 Da |
| Oxidation | +15.99 Da |
| Cation: Na | +21.98 Da |
| Dimethylation | +28.03 Da |
| Cation: K | +37.96 Da |
| Acetylation | +42.01 Da |
| Carbamylation | +43.01 Da |
| Cation: Fe[III] | +52.91 Da |
| Cation: Fe[II] | +53.92 Da |
| Phosphorylation | +79.97 Da |
| Aminoethylbenzenesulfonylation (AEBs) | +183.04 Da |

**Table S11**: Parameter settings of ProMex

| Parameter | Value |
| --- | --- |
| Min charge | 1 |
| Max charge | 30 |
| Min mass | 1,000 Da |
| Max mass | 70,000 Da |
| m/z error tolerance of spectral peaks | 16 ppm |
| Likelihood score threshold | -10 |

**Table S12**: Parameter settings of MSPathFinder

| Parameter | Value |
| --- | --- |
| Internal cleavage search mode | Single internal cleavage |
| Tag-based search | True |
| # of matches to keep in memory | 3 |
| Target and shuffled decoy database | True |
| Precursor tolerance | 10 ppm |
| Fragment ion tolerance | 10 ppm |
| Min sequence length | 21 |
| Max sequence length | 500 |
| Min charge | 1 |
| Max charge | 30 |
| Min fragment ion charge | 1 |
| Max fragment ion charge | 30 |
| Min sequence mass | 3,000 Da |
| Max sequence mass | 70,000 Da |

**Figures**


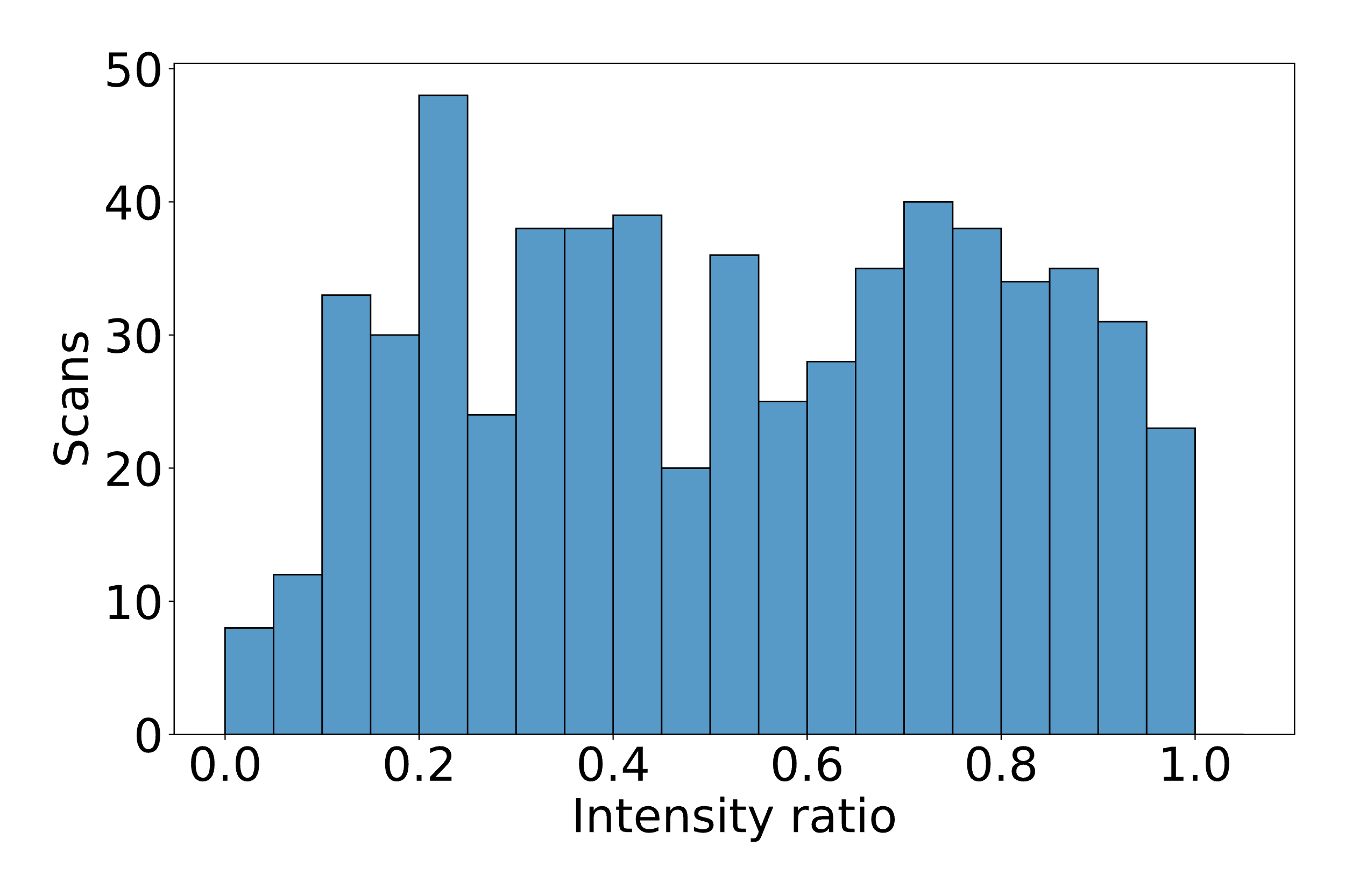


**Fig. S1**: The distribution of the intensity ratio of the second and first precursors in the 615 MS/MS spectra with proteoform pair identifications reported from the first replicate of the yeast dataset


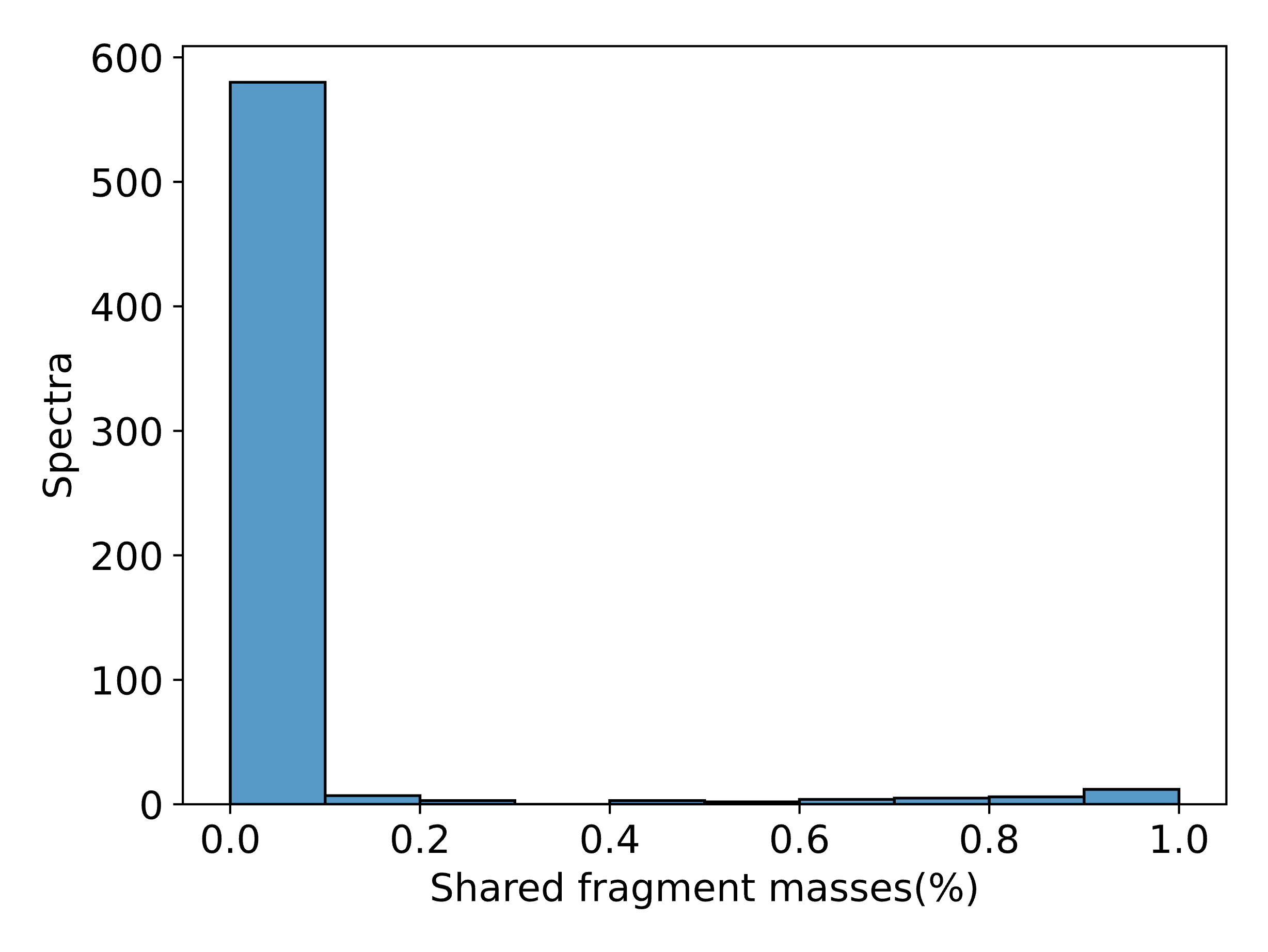


**Fig. S2**: Distribution of the ratios of shared matched fragment masses in the PrSM pairs of the 622 spectra reported from the first replicate of the yeast dataset


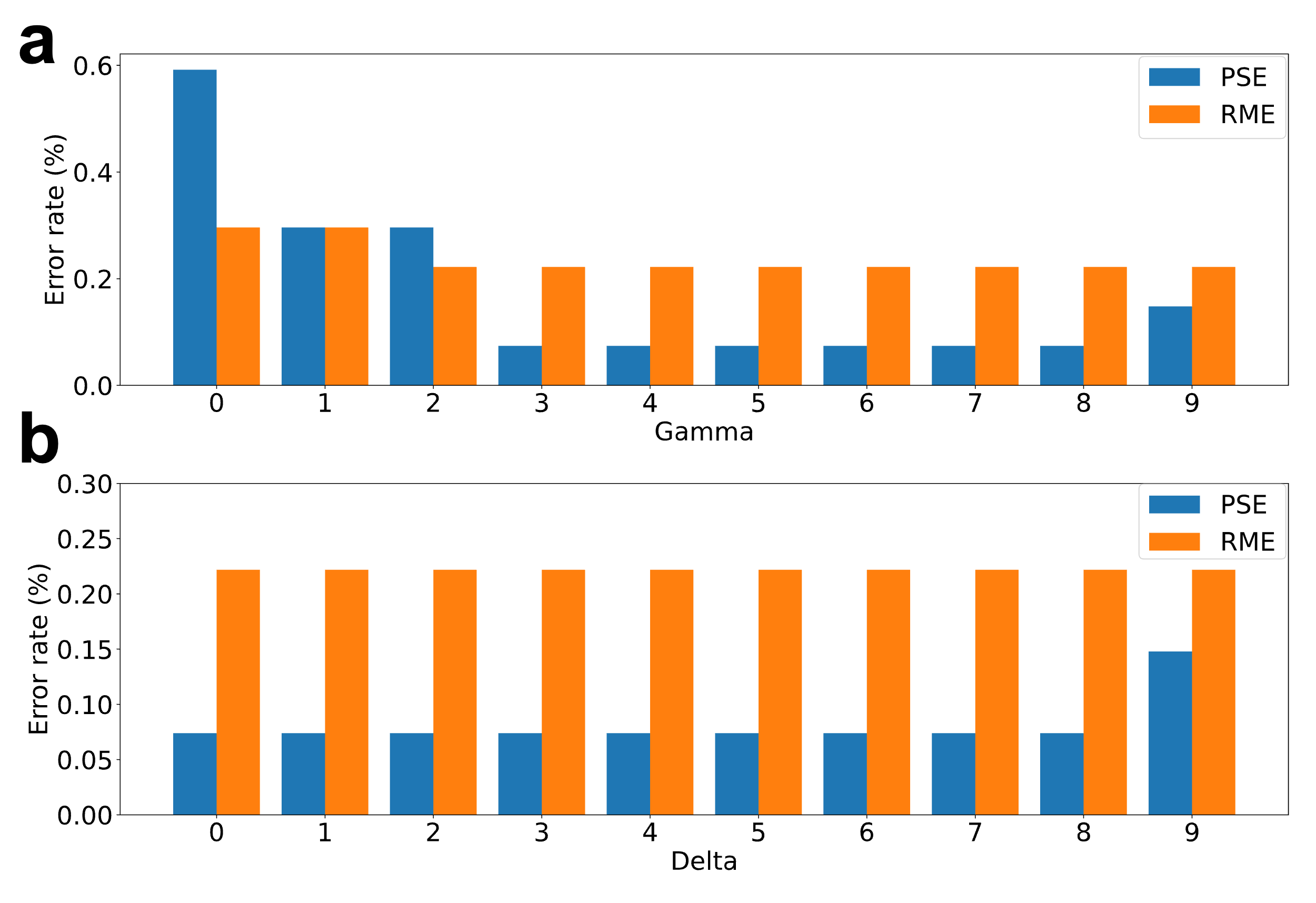


**Fig. S3: PSE and RME rates of TopMPI with various *δ* and *γ* parameter settings on the RPMS dataset generated with an A-B ratio of 100%. (a)** Error rates with ***δ* = 5** and varying settings for ***γ*** (**0, 1, ..., 9**). **(b)** Error rates with ***γ* = 4** and varying settings for ***δ*** (**0, 1, ..., 9**).


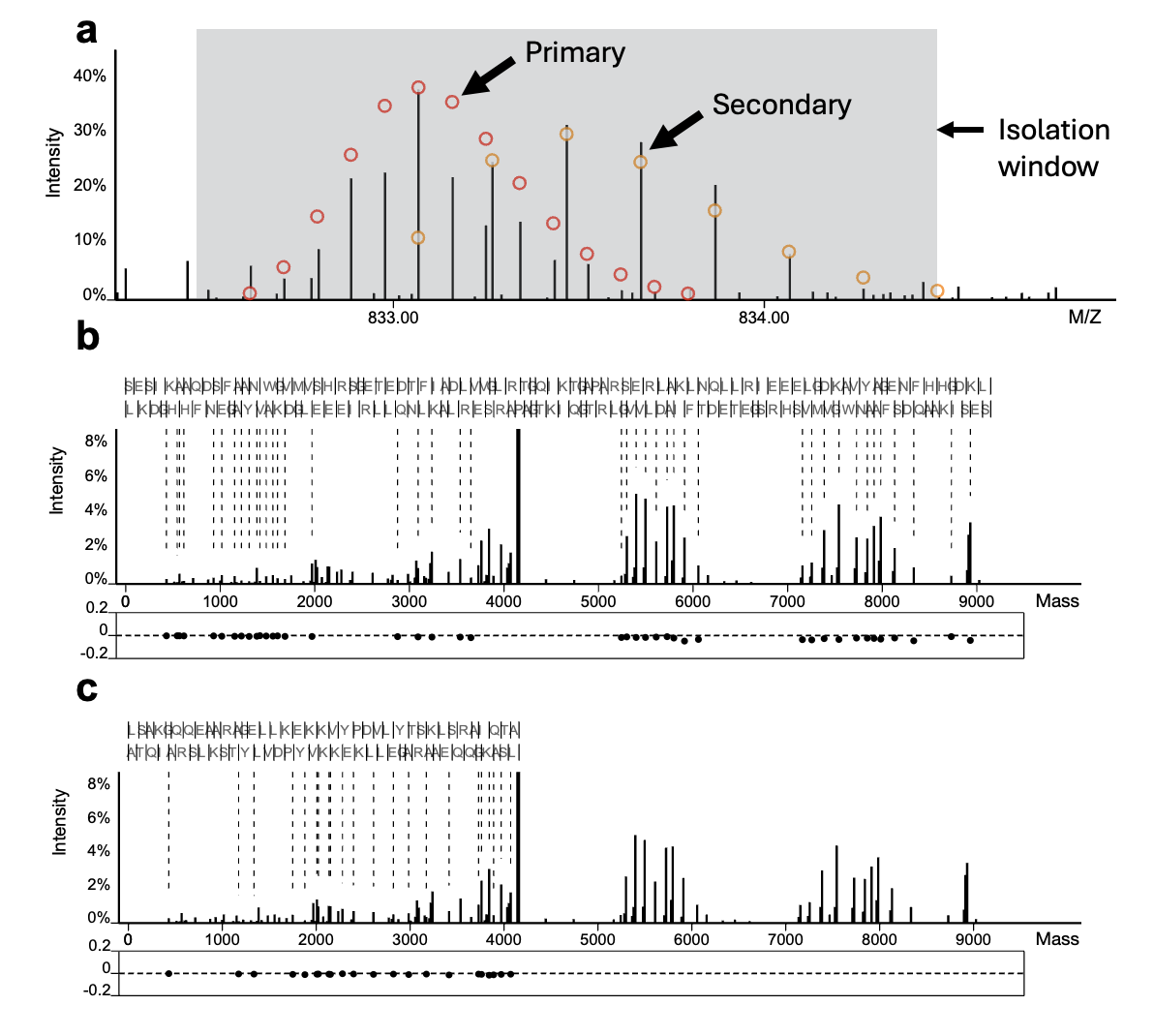


**Fig. S4**: Annotations of the precursor ions and fragment masses of scan 2450 of the first replicate of the yeast dataset. (a) Primary and secondary precursors in the isolation window. (b) Matched fragment masses in the PrSM of the primary precursor. (c) Matched fragment masses in the PrSM of the secondary precursor.


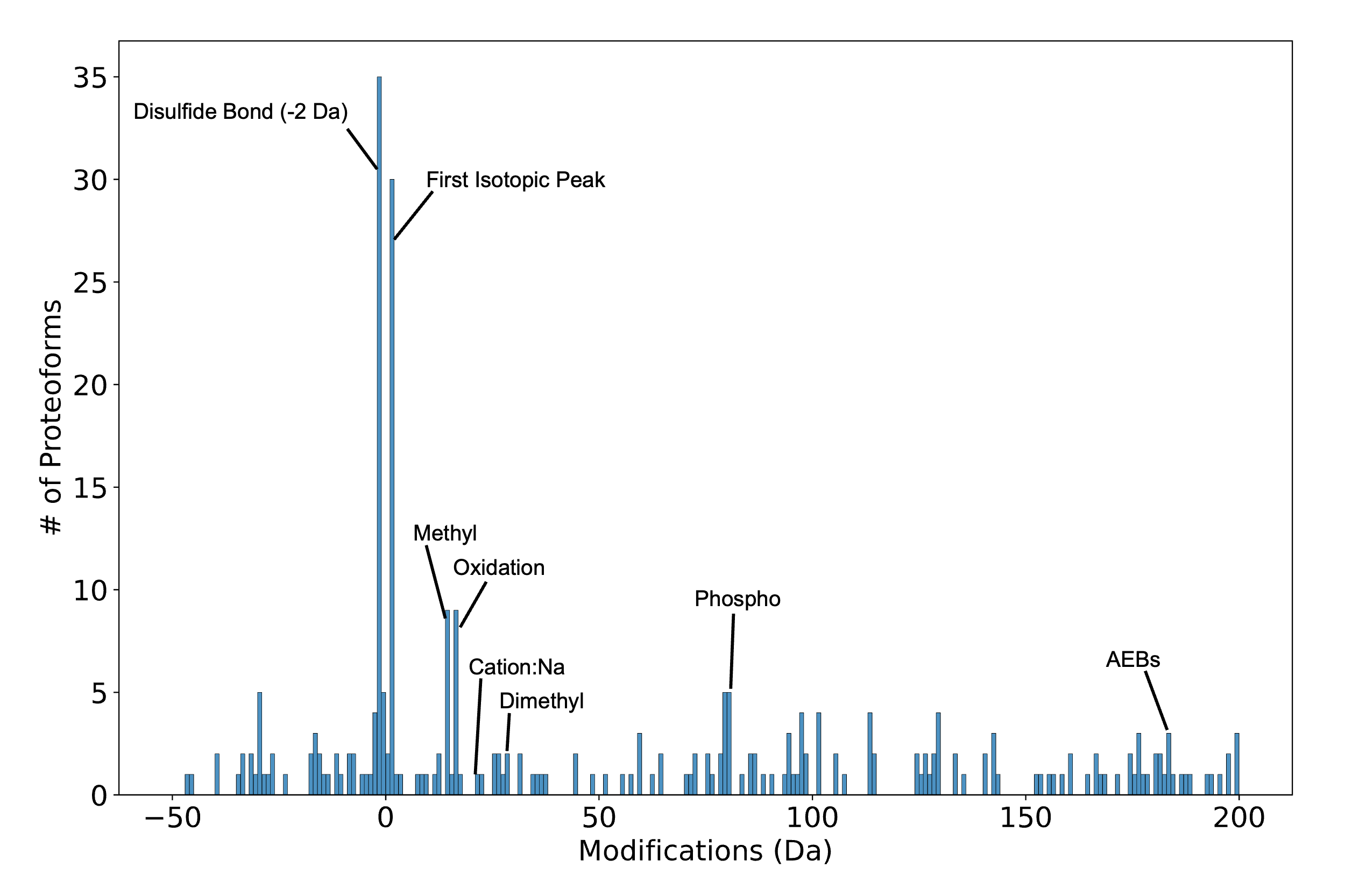


**Fig. S5**: Histogram of the mass shifts in proteoforms identified from primary precursors only by TopMPI from the first replicate of the yeast dataset

**
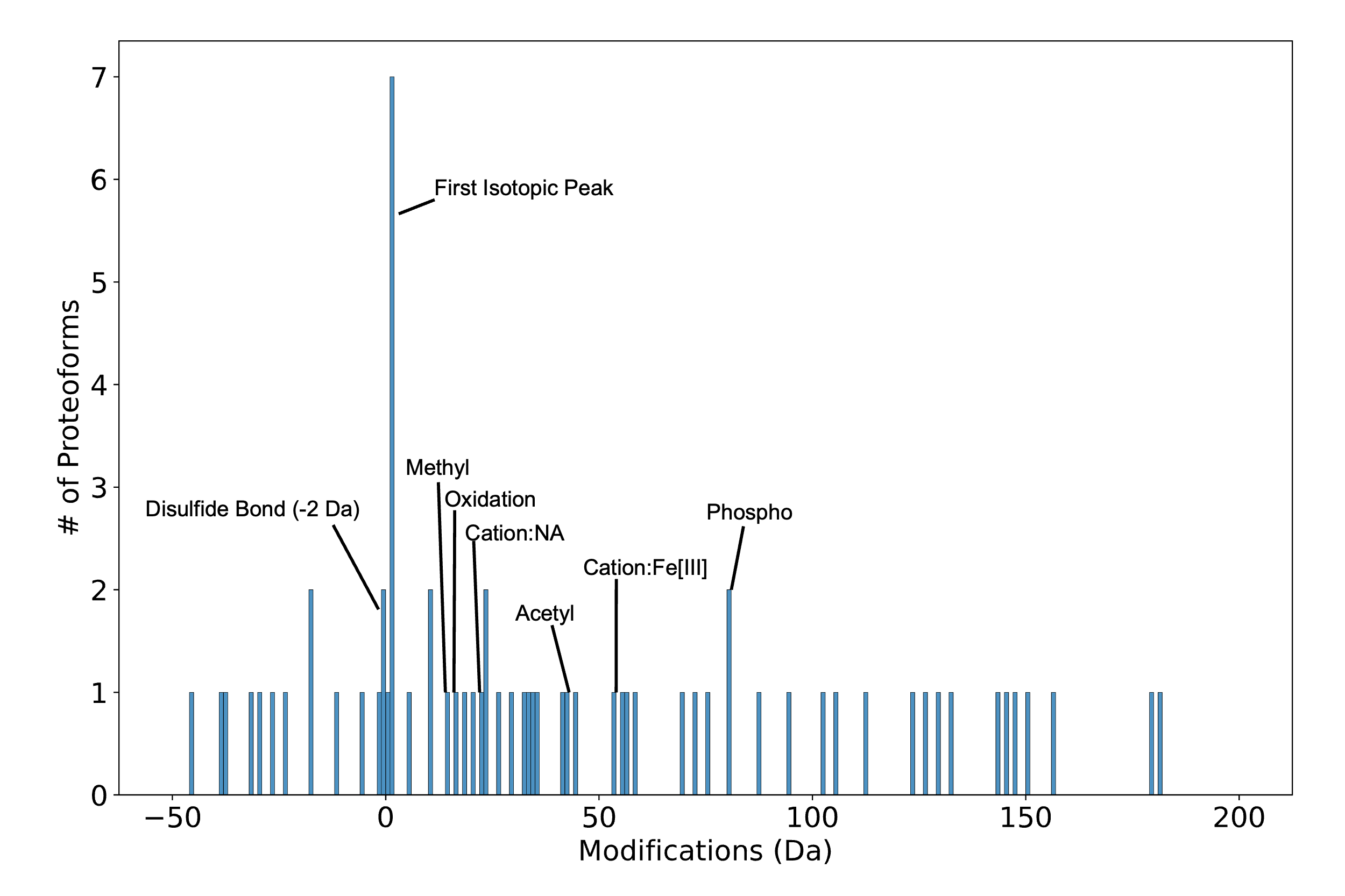
**

**Fig. S6**: Histogram of the mass shifts in proteoforms identified from secondary precursors only by TopMPI from the first replicate of the yeast dataset


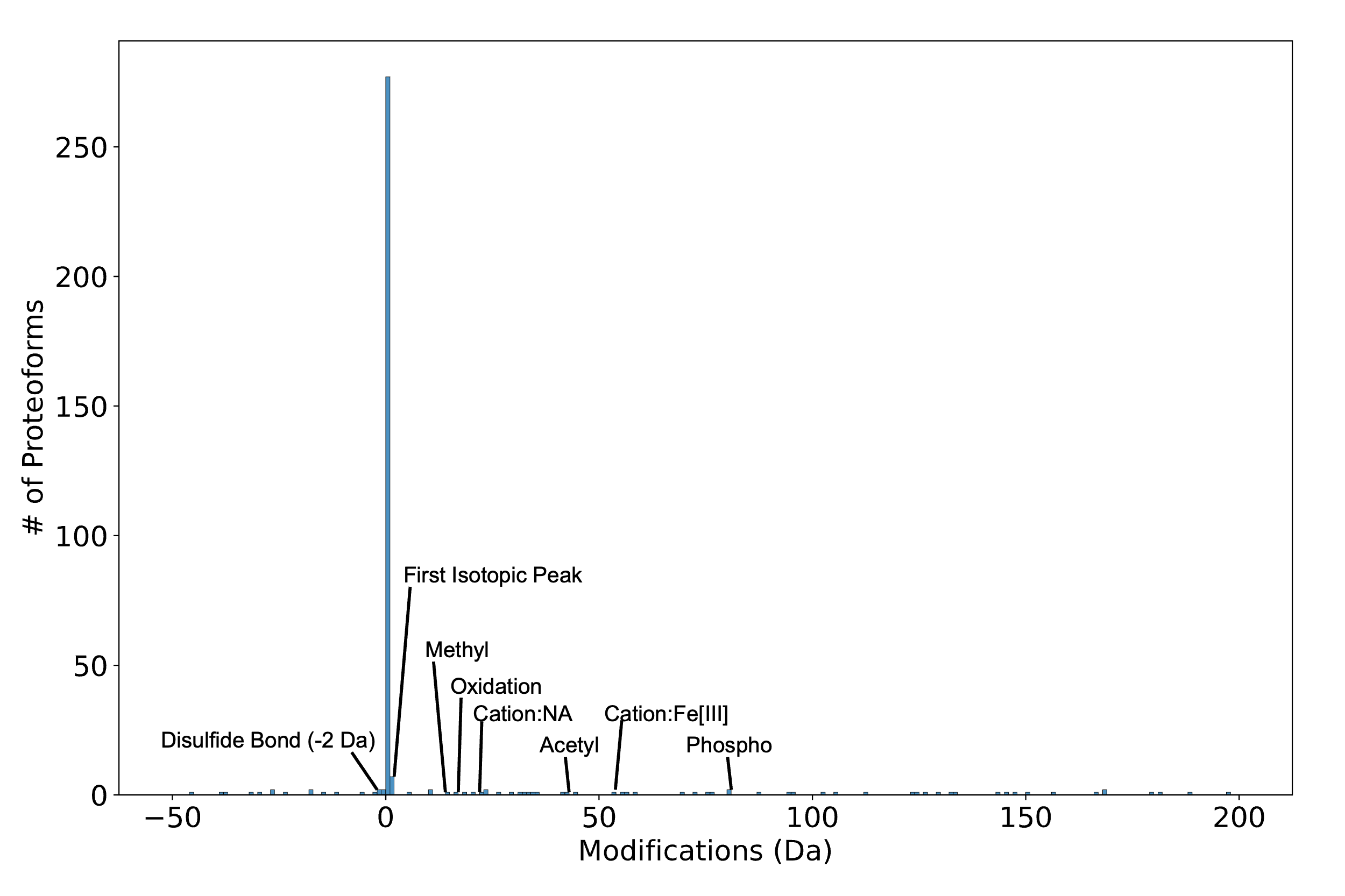


**Fig. S7**: Histogram of the mass shifts in the proteoforms identified by TopMPI but missed by TopPIC from the first replicate of the yeast dataset


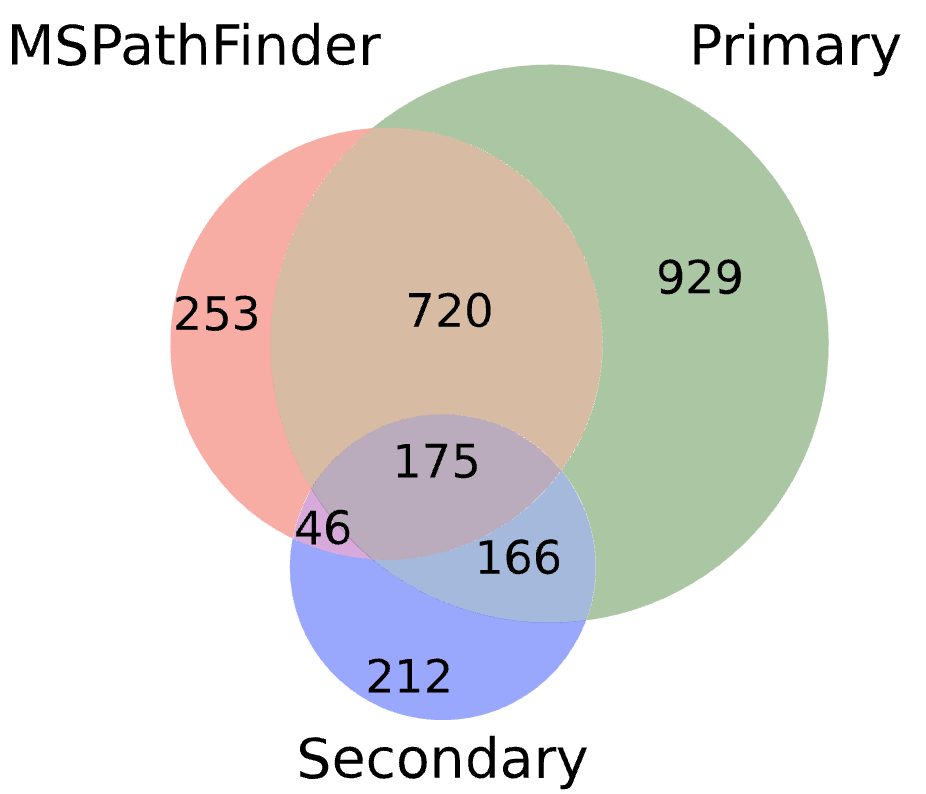


**Fig. S8**: Comparison of proteoform identifications reported by MSPathFinder and by TopMPI from primary and secondary precursors from the first replicate of the yeast dataset
